# TASK-SPECIFIC METACOGNITIVE ACCURACY DECLINES ACROSS THE DEMENTIA-ALZHEIMER’S TYPE SPECTRUM

**DOI:** 10.1093/geroni/igz038.003

**Published:** 2019-11-08

**Authors:** Annalise M Rahman-Filipiak, Arijit Bhaumik, Bruno Giordani, Henry Paulson, Benjamin M Hampstead

**Affiliations:** 1 University of Michigan, Ann Arbor, United States; 2 Michigan Alzheimer’s Disease Center, Ann Arbor, Michigan, United States; 3 University of Michigan, Ann Arbor, Michigan, United States

## Abstract

Subjective cognitive complaints (SCCs) remain part of the diagnostic criteria for amnestic mild cognitive impairment (aMCI), the prodromal stage of dementia - Alzheimer’s type (DAT), despite weak relationships between self-reported and objectively-measured functioning. Most metacognitive measures focus on ratings of global retrospective memory rating only; greater subtlety in measurement of SCCs is required. Similarly, it is critical to identify the disease stage at which the clinical utility of SCCs is nullified by impaired insight. This study aims to evaluate group differences in (a) task-specific metacognitive ratings, and (b) the accuracy of these ratings in individuals diagnosed as cognitively intact (CI), with aMCI, or with DAT. 99 older adults (M-age = 69.43, SD-age = 6.98; M-edu = 15.54, SD-edu = 2.47; CI: n = 50, aMCI: n = 34, DAT: n = 15) enrolled in the University of Michigan Memory and Aging Project rated their performance on the Object Location Touchscreen Task (OLTT), an ecologically valid memory measure. One-way analysis of variance (ANOVA) revealed that individuals with aMCI-multiple domain or DAT rated their memory performance similarly to CI individuals, though the aMCI-single domain group rated themselves as more impaired. Bivariate Pearson’s r correlations demonstrated a decline in the strength of the relationship between task-specific metacognitive ratings and actual OLTT memory performance with increasing diagnostic severity. These findings suggest a decline in insight on task-specific memory ratings across the DAT spectrum, and call into question the use of self-reported SCCs as a diagnostic tool in later stages of disease progression.

